# Supplementary material for: Synthesis of ultra-high molecular weight homo- and copolymers via an ultrasonic emulsion process with a fast rate
Source: Commun Chem. 2024 May 16;7:113. doi: 10.1038/s42004-024-01191-6 (PMC11099186; doi:10.1038/s42004-024-01191-6)
Supplement: Supplementary file 2 — Supplementary Information [file 42004_2024_1191_MOESM2_ESM.pdf]

Supplementary Information

Synthesis of ultra-high molecular weight homo- and copolymers *via* an ultrasonic emulsion process with a fast rate

Uddhab Kalita[a,b,c], Vianna F. Jafari[a], Muthupandian Ashokkumar[b], Nikhil K. Singha[c]*, Greg G. Qiao[a]*

[a] *Polymer Science Group, Department of Chemical Engineering, The University of Melbourne, Parkville 3010, Victoria, Australia*
[b] *School of Chemistry, The University of Melbourne, Parkville 3010, Victoria, Australia*
[c] *Rubber Technology Centre, Indian Institute of Technology Kharagpur, Kharagpur 721302, WB, India*

*Corresponding authors' email IDs: gregghq@unimelb.edu.au, nks@rtc.iitkgp.ac.in

ORCID ID:

U Kalita: https://orcid.org/0000-0002-0187-7782
V.F. Jafari: https://orcid.org/0000-0003-1308-1754
M. Ashokkumar: https://orcid.org/0000-0002-8442-1499
N. K. Singha: https://orcid.org/0000-0003-0935-0157
G. G. Qiao: http://orcid.org/0000-0003-2771-9675

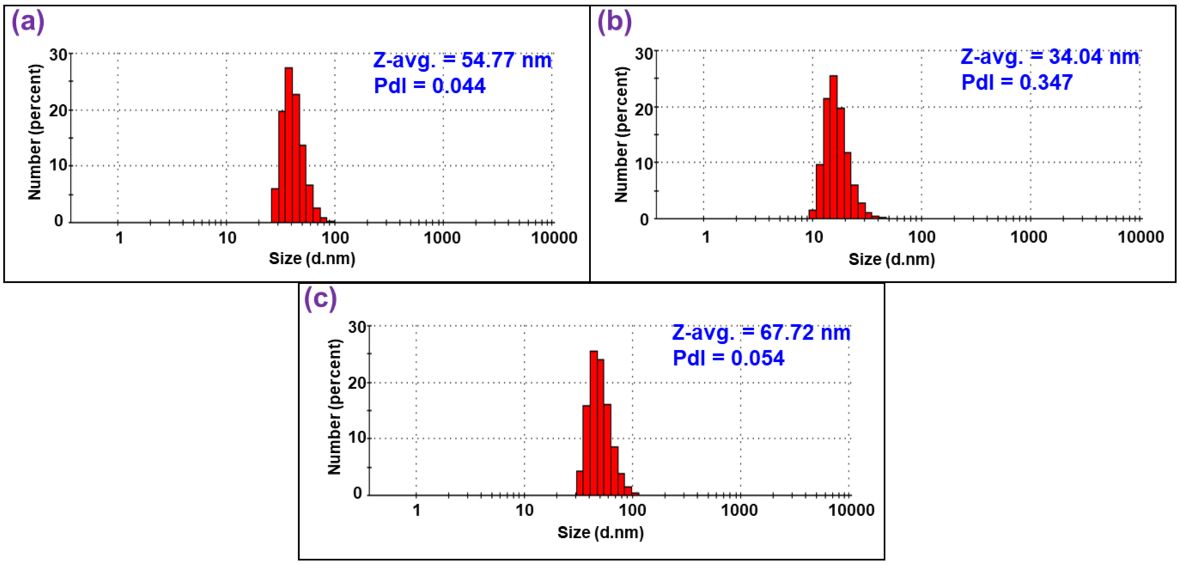

Supplementary Figure 1: Particle size distribution of (a) PBMA (H1), (b) PMMA (H3), (c) PHA (H2) latexes obtained using DLS.

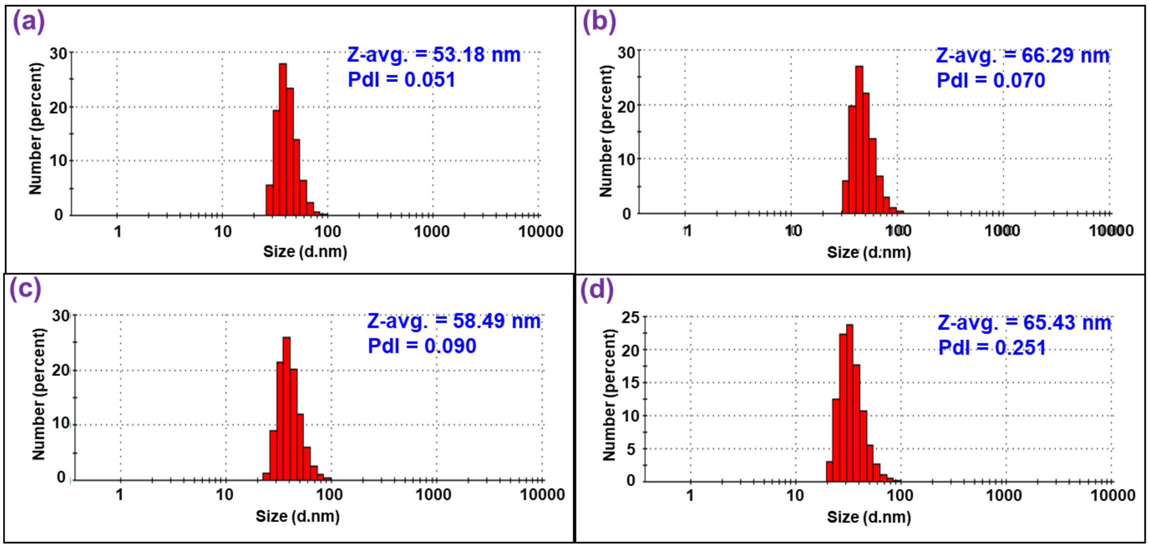

Supplementary Figure 2: DLS analysis of the latexes and their Z-avg. (d.nm) and PdI values after 4 months of polymerization for (a) PBMA (H1), (b) PHA (H2), (c) PIBMA (H4), (d) PLA (H5) after 4 months.

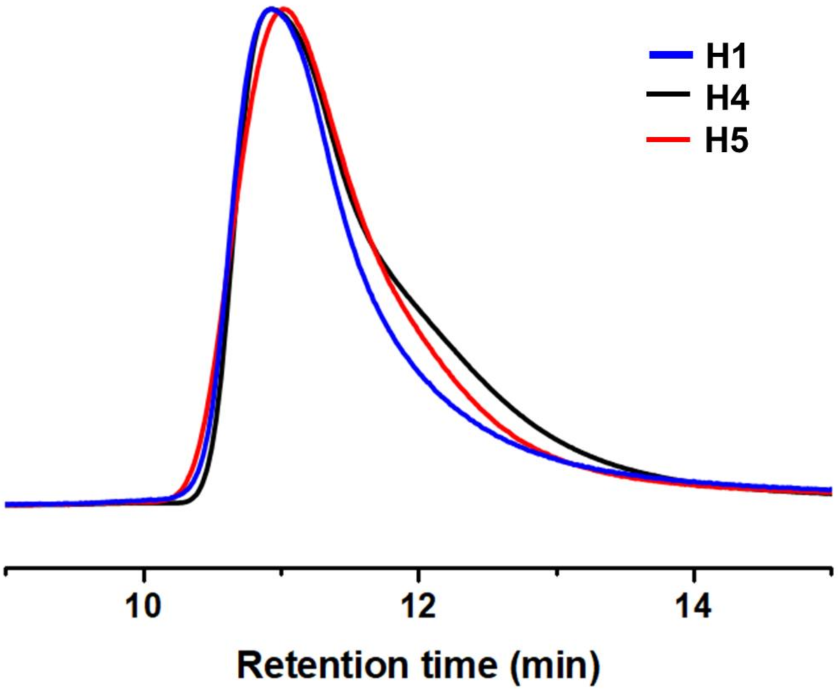

Supplementary Figure 3: GPC traces of the homopolymers of BMA (**H1**), IBMA (**H4**), and LA (**H5**) prepared using ultrasound-initiated emulsion polymerization.

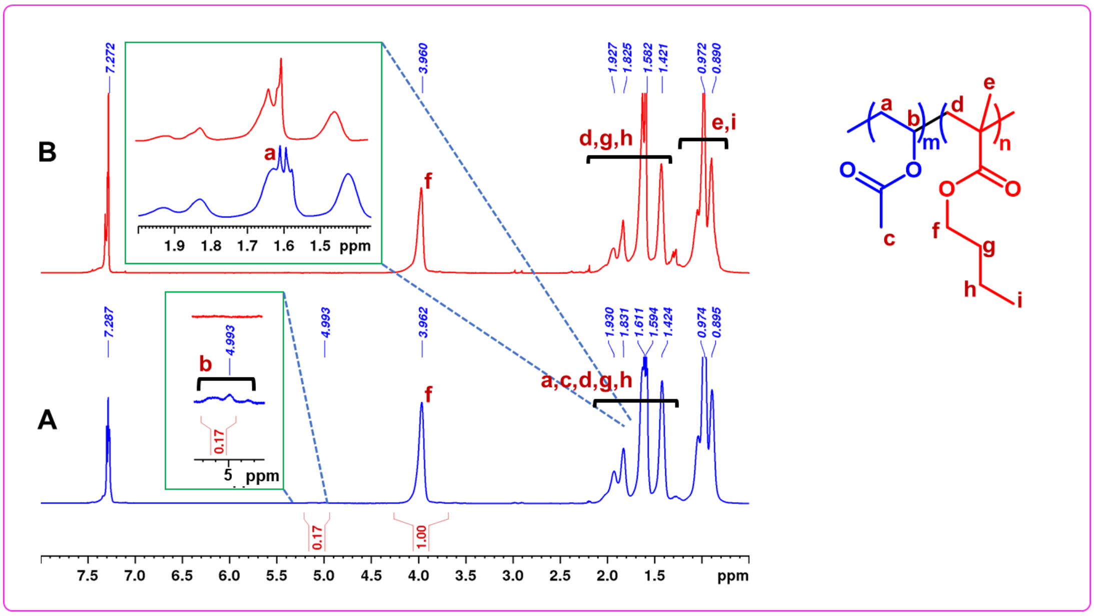

Supplementary Figure 4: ^{1}H NMR of A) $PVAc_{50}$-co-$PBMA_{50}$ (C5) and B) PBMA (H1) in $CDCl_3$. The emergence of new peaks at about 1.6 ppm and 5 ppm is for the "a" and "b" protons, respectively of the vinyl acetate component of the copolymer. The same were not present for the ^{1}H NMR of the PBMA homopolymer and thus proves the fact that the C5 polymer is a copolymer of BMA and VAc and not a homopolymer of BMA. The integration area under the curve for proton "b" of the VAc component, and proton "f" of the BMA component as shown in the figure also agree with the experimental conversion (65 wt. %) achieved as listed in Table 2 of the main text.

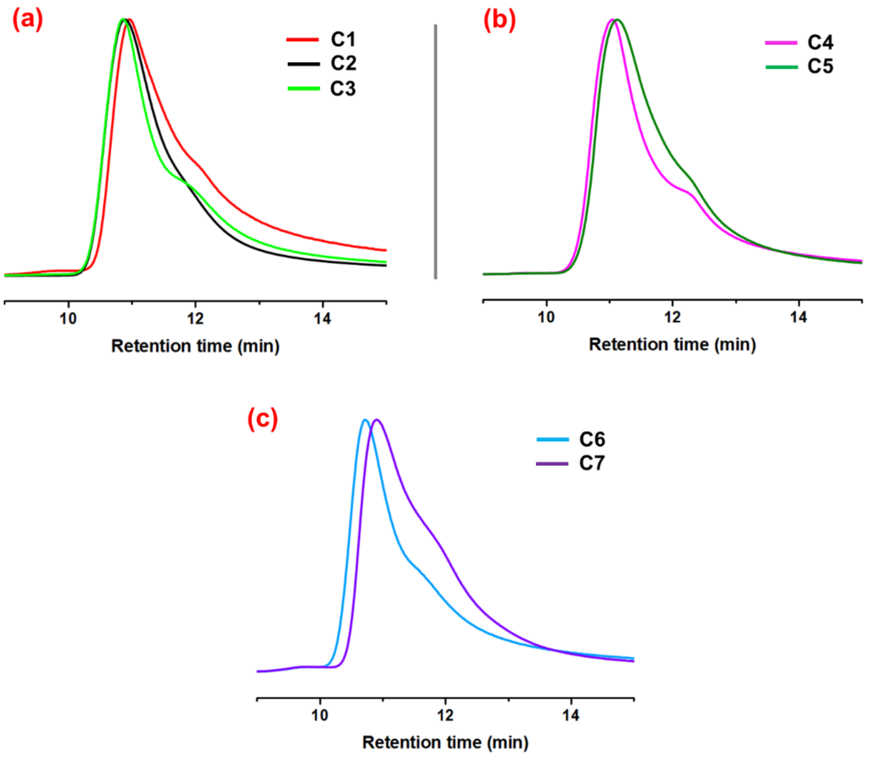

Supplementary Figure 5: GPC traces of the (a) (meth)acrylate di-copolymers, (b) Vinyl acetate-based copolymers, and (c) tri-copolymers prepared using ultrasound-initiated emulsion polymerization. The different copolymers are labelled as follows: C1 = Poly(MMA-*co*-BMA); C2 = Poly(IBMA-*co*-BMA); C3 = Poly(LA-*co*-BMA); C4 = Poly(VAc_{20}-*co*-BMA_{80}); C5 = Poly(VAc_{50}-*co*-BMA_{50}); C6 = Poly(BMA-*co*-IBMA-*co*-LA); C7 = Poly(MMA-*co*-BMA-*co*-IBMA).

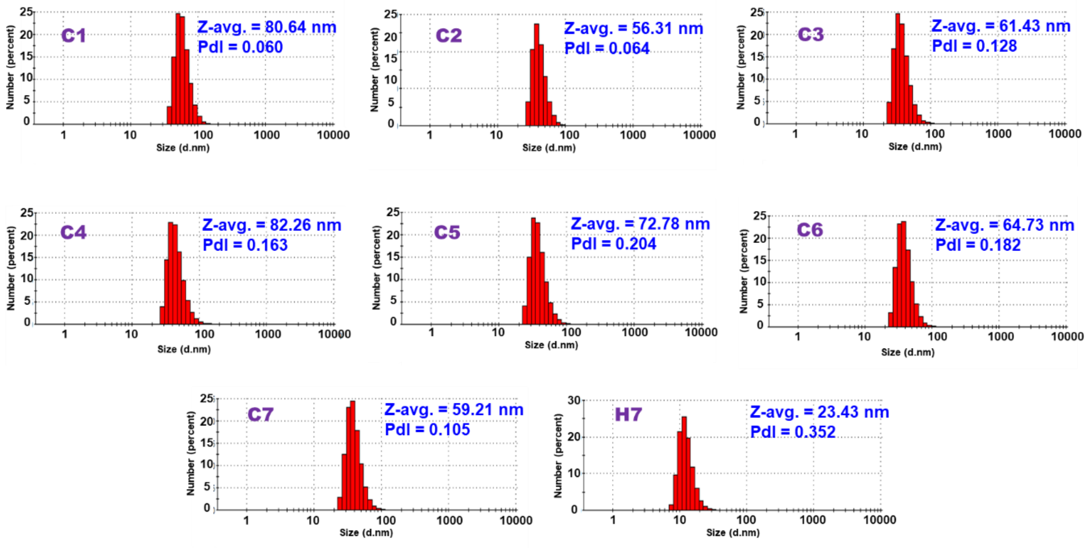

Supplementary Figure 6: DLS analysis of the different copolymers, and H7. The different copolymers are labelled as follows: C1 = Poly(MMA-co-BMA) ; C2 = Poly(IBMA-*co*-BMA); C3 = Poly(LA-*co*-BMA); C4 = Poly(VAc_{20}-*co*-BMA_{80}); C5 = Poly(VAc_{50}-*co*-BMA_{50}); C6 = Poly(BMA-*co*-IBMA-*co*-LA); C7 = Poly(MMA-*co*-BMA-*co*-IBMA). H7 is PBMA prepared using semi bio-Fenton chemistry.

Supplementary Equation 1: Fox equation:

$$\frac{1}{T_g} = \frac{w_1}{T_{g,1}} + \frac{w_2}{T_{g,2}}$$

where,

T_g represents the glass transition temperature (K) of the copolymer of component 1 & 2
$T_{g,1}$ represents the glass transition temperature (K) of the homopolymer of component 1
$T_{g,2}$ represents the glass transition temperature (K) of the homopolymer of component 2
w_1 and w_2 are weight fractions of the components 1 & 2, respectively in the feed mixture

Using this equation, the T_g of a copolymer can be theoretically predicated if the T_g of the homopolymers of the other components are known. As for example, the T_g of H1 and H4 are 38.0 °C and 161.5 °C, then using the Fox equation, the theoretical value for the copolymer C2 can be found as follows:

$$\frac{1}{T_g} = \frac{w_1}{T_{g,1}} + \frac{w_2}{T_{g,2}}$$

$$\frac{1}{T_g} = \frac{0.5}{(38 + 273)} + \frac{0.5}{(161.5 + 273)}$$

$$\therefore T_g = 362.5\ K$$

$$\therefore T_g = 89.5\ ℃$$

The experimentally recorded value of 85.8 °C for the same copolymer (**C2**) is in close accordance with the value obtained theoretically (89.5 °C) from the Fox equation.

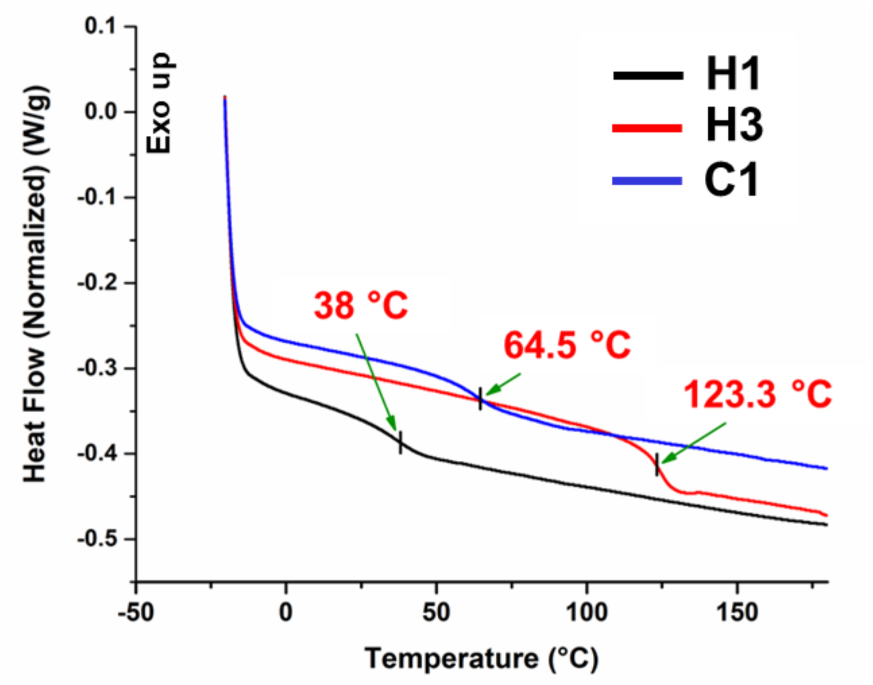

Supplementary Figure 7: DSC traces of PBMA (**H1**), PMMA (**H3**) and poly(MMA-*co*-BMA) (**C1**). The single glass transition temperatures obtained for the copolymers solidify that a truly random copolymer has been produced after polymerization rather than forming different blocks or blends of the same monomers.

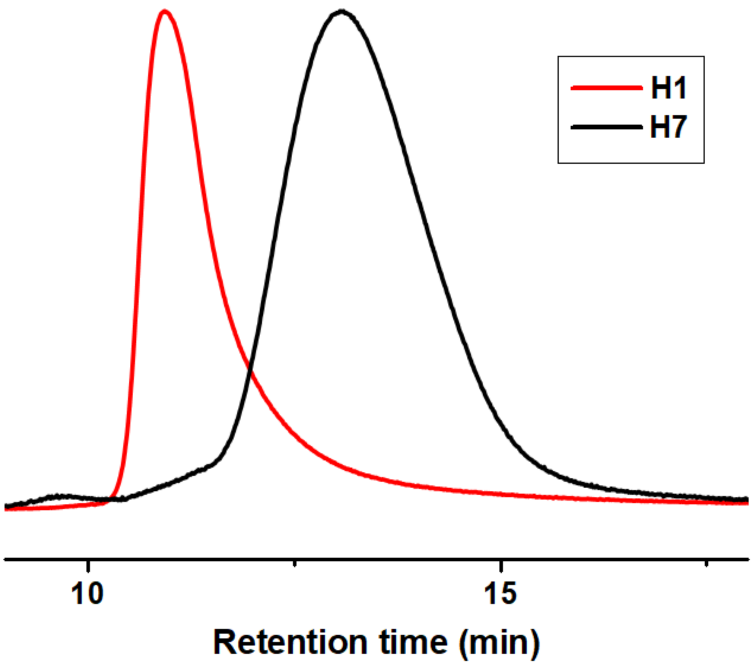

Supplementary Figure 8: GPC traces of homopolymers of BMA prepared using ultrasound initiated (H1) and semi bio-Fenton chemistry based (H7) emulsion systems.

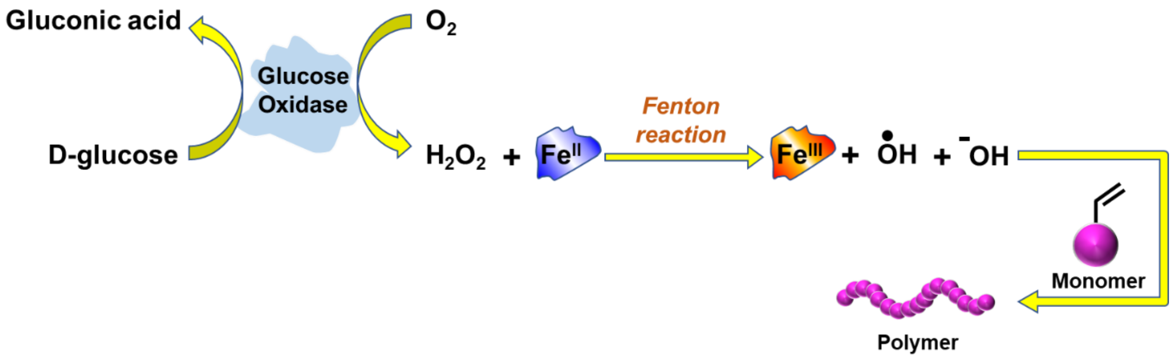

Supplementary Figure 9: General schematic illustration of the Semi Bio-Fenton polymerization in the presence of air. Glucose oxidase (GOx) oxidizes D-glucose to gluconic acid in the presence of molecular oxygen (air), in turn producing H_2O_2. The produced H_2O_2 then reacts with Fe^{2+} (Ammonium ferrous sulfate) *via* the Fenton chemistry generating hydroxyl radicals (•OH) which in turn can be utilized for polymerization reaction.

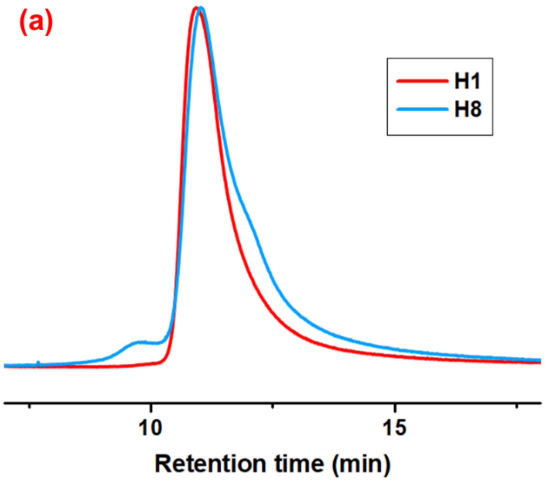

(b)

Sample ID	M_n (g/mol)	Đ
H1	7.60×10^6	1.01
H8	5.30×10^6	1.10

Supplementary Figure 10: (a) GPC traces of homopolymers of BMA prepared using ultrasonic initiation (**H1**) and Enzyme catalyzed deoxygenation combined with ultrasonic initiation (**H8**) emulsion systems. (b) Table representing the data for H1 and H8 PBMA homopolymers.

Enzyme catalyzed system to remove the dissolved oxygen from the polymerization system instead of argon to purge it, was also employed before applying US to the reaction mixture for initiation. GOx enzyme in presence of oxygen can turn D-glucose into hydrogen peroxide and gluconic acid, eventually removing the oxygen out of the system. Consequently, chemistry involved with these two reagents was employed to remove dissolved oxygen from the polymerization system before applying US to it for initiation of polymerization. However, the gluconic acid produced during this process may disrupt the emulsion and thus to maintain the

pH, a little bit of $NaHCO_3$ was added to the system. The water, SDS, and monomer ratio was maintained same as that followed for H1 polymerization. The MW achieved was comparable (5.3×10^6 g/mol) what was achieved using argon to purge the system with a slightly broader dispersity of 1.10 (**H8**, **Supplementary Figure 10**). However, the time required for a full conversion increased from 15 minutes to 1 hour. It was established previously that dissolved gases play a great role in the flux of radicals generated during ultrasonication.[1] Having argon in the polymerization system, enhances the cavitation bubble collapse intensity which is probably not of the same intensity for a system where oxygen is removed by means of an enzyme catalyzed system. Thus, a slower polymerization rate was observed compared to the argon purged system.

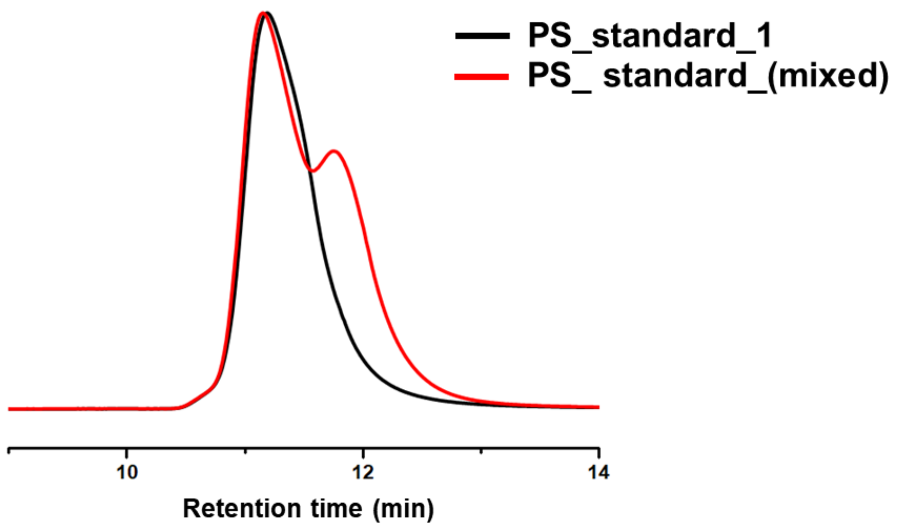

Supplementary Figure 11: GPC traces of polystyrene standard samples. The PS_standard_1 represents a standard sample with Đ of 1.01 and MW of 2.9×10^6 g/mol. The other GPC trace designated as PS_standard_mix represents a mixture of two polystyrene standard samples, where the higher MW PS_standard_1 has been mixed with a comparatively lower MW of about 1 million with Đ of 1.01. The GPC trace clearly shows a bimodal distribution establishing the fact that the columns and MALS detector used throughout this study are trustworthy and are able to sperate even higher MW polymers.

(a)

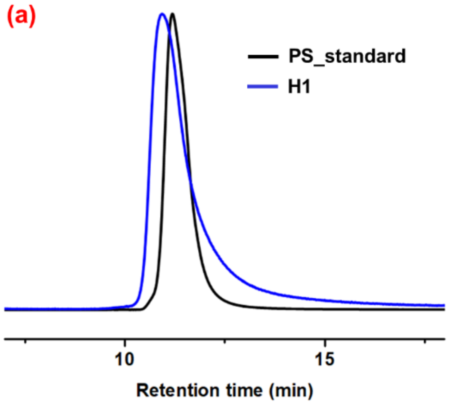

(b)

Sample ID	$M_n^{\#}$ (g/mol)	Đ
H1 (PBMA)	7.60×10^{6}	1.01
PS_standard_sample	2.90×10^{6}	1.01

Supplementary Figure 12: (a) GPC trace of homopolymer of BMA (**H1**) prepared using ultrasonic initiation (**H1**) and a polystyrene standard sample (PS_standard) used for calibration of GPC instruments with a reported Đ of 1.01. Both the traces are recorded using the same instrument, detector, and software used throughout this work. (b) The table displays the MW and the Đ for the respective homopolymers. As the GPC trace of H1 aligns with the GPC trace of the standard sample, while considering the data displayed in the Table, it can be concluded that the column and the MALS detector utilized along with the ASTRA software used during this work are good enough for determination of MW and Đ for the UHMW polymers as well.

Supplementary Table 1: dn/dc values used for the different homo- and co- polymers during this work. The value was entered into the ASTRA software while obtaining the GPC data utilizing the static light scattering detector.

**Polymer** $^{\Psi}$	**dn/dc**
PBMA	0.0750
PHA	0.1000
PMMA	0.0810
PIBMA	0.1000
PLA	0.0700
Poly(MMA-*co*-BMA)	0.0800
Poly(IBMA-*co*-BMA)	0.0800
Poly(LA-*co*-BMA)	0.0800
Poly(VAc_{20}-*co*-BMA_{80})	0.0600
Poly(VAc_{50}-*co*-BMA_{50})	0.0600
Poly(BMA-*co*-IBMA-*co*-LA)	0.0900
Poly(MMA-*co*-BMA-*co*-IBMA)	0.0800

$^{\Psi}$ The numbers 20, 50 & 80 represent the feed ratio (wt. %) of the particular monomer into the polymerization system.

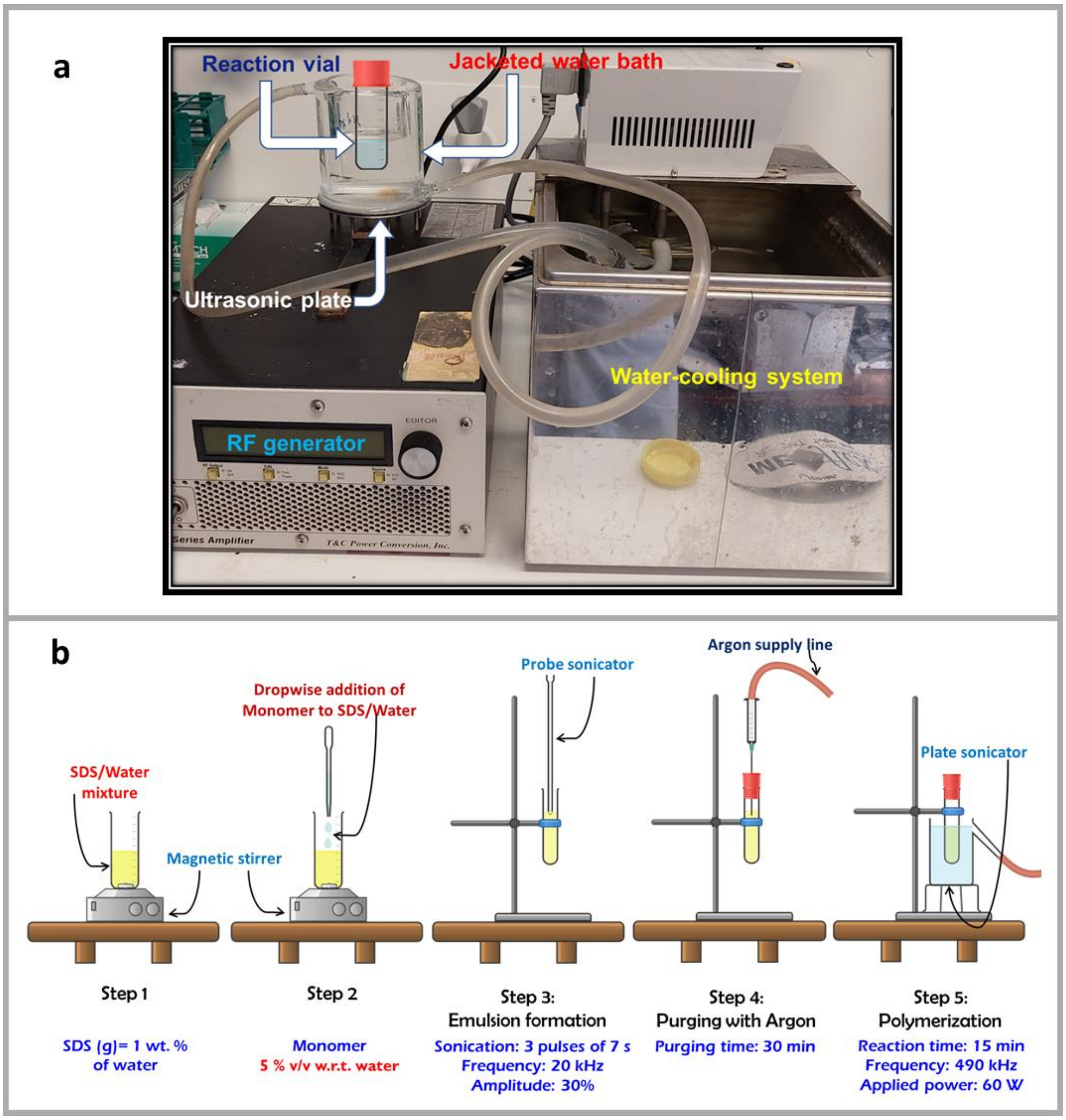

Supplementary Figure 13: (a) Step-by-step method applied for polymerization of the homo- and co- polymerization of various hydrophobic monomers using ultrasound-initiated emulsion polymerization technique. **(b)** Image of the ultrasonic set-up used for all the experiments.

Supplementary References:

1. Santha Kumar, A. R. S. *et al*. Ultrasonics in polymer science: applications and challenges. *Prog. Mater. Sci.* **136**, 101113 (2023).
