# Supplementary material for: Synthesis of ultra-high molecular weight homo- and copolymers via an ultrasonic emulsion process with a fast rate
Source: Commun Chem. 2024 May 16;7:113. doi: 10.1038/s42004-024-01191-6 (PMC11099186; doi:10.1038/s42004-024-01191-6)
Supplement: Supplementary file 3 — Description of Additional Supplementary Files [file 42004_2024_1191_MOESM3_ESM.pdf]

Description of Additional Supplementary Files

File name: Supplementary Data 1
Description: ^{1}H NMR of PBMA (H1) and Poly(VAc_{50}-*co*-BMA_{50}) (C5).

File name: Supplementary Data 2
Description: GPC trace data.

File name: Supplementary Data 3
Description: DSC curve data.

File name: Supplementary Data 4
Description: TGA curve data.
