# Supplementary material for: Synthesis of ultra-high molecular weight homo- and copolymers via an ultrasonic emulsion process with a fast rate
Source: Commun Chem. 2024 May 16;7:113. doi: 10.1038/s42004-024-01191-6 (PMC11099186; doi:10.1038/s42004-024-01191-6)
Supplement: Supplementary file 4 — Supplementary Data 1 [file 42004_2024_1191_MOESM4_ESM.pdf]

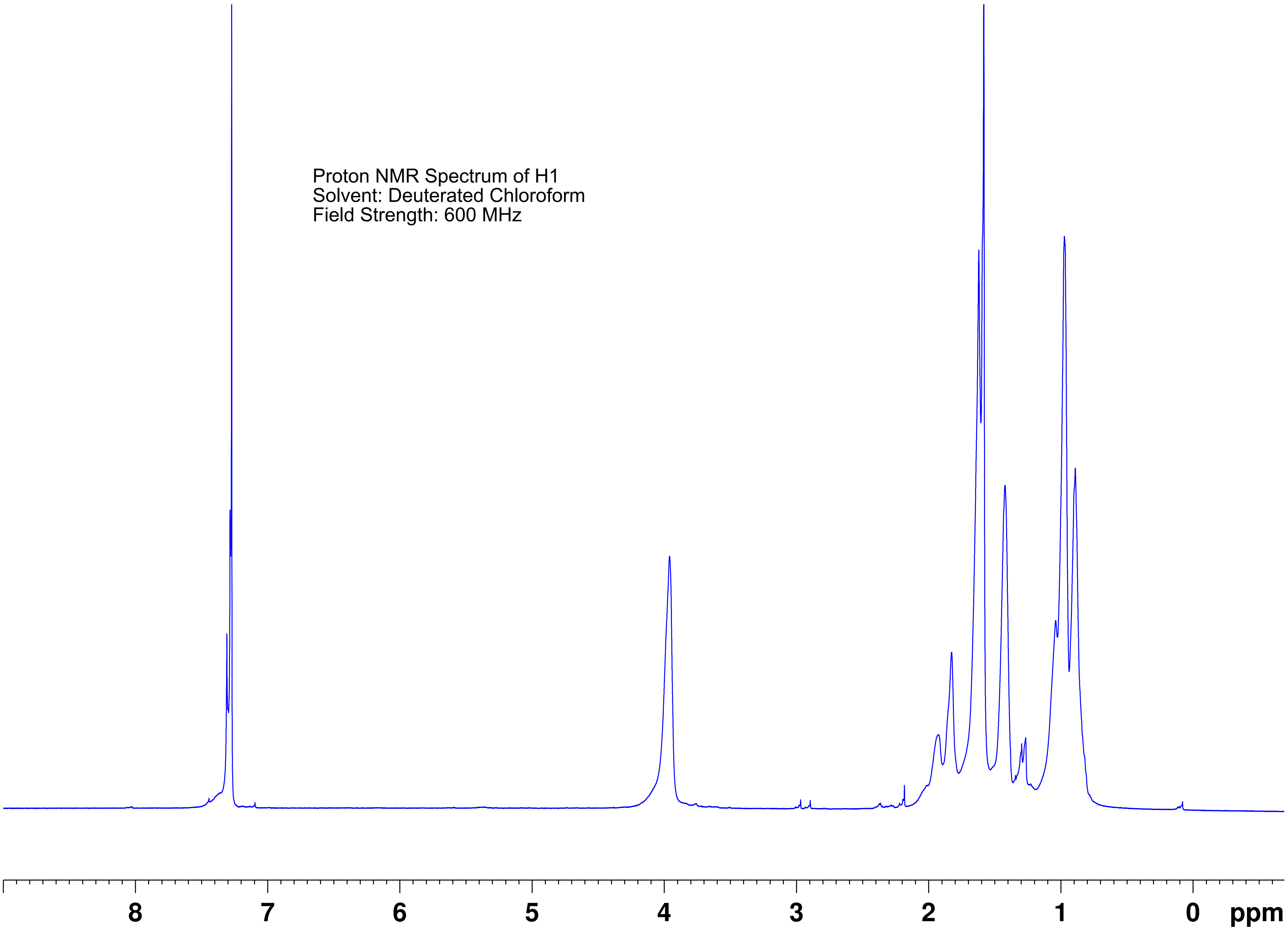
Proton NMR Spectrum of H1
Solvent: Deuterated Chloroform
Field Strength: 600 MHz
8
7
6
5
4
3
2
1
0
ppm

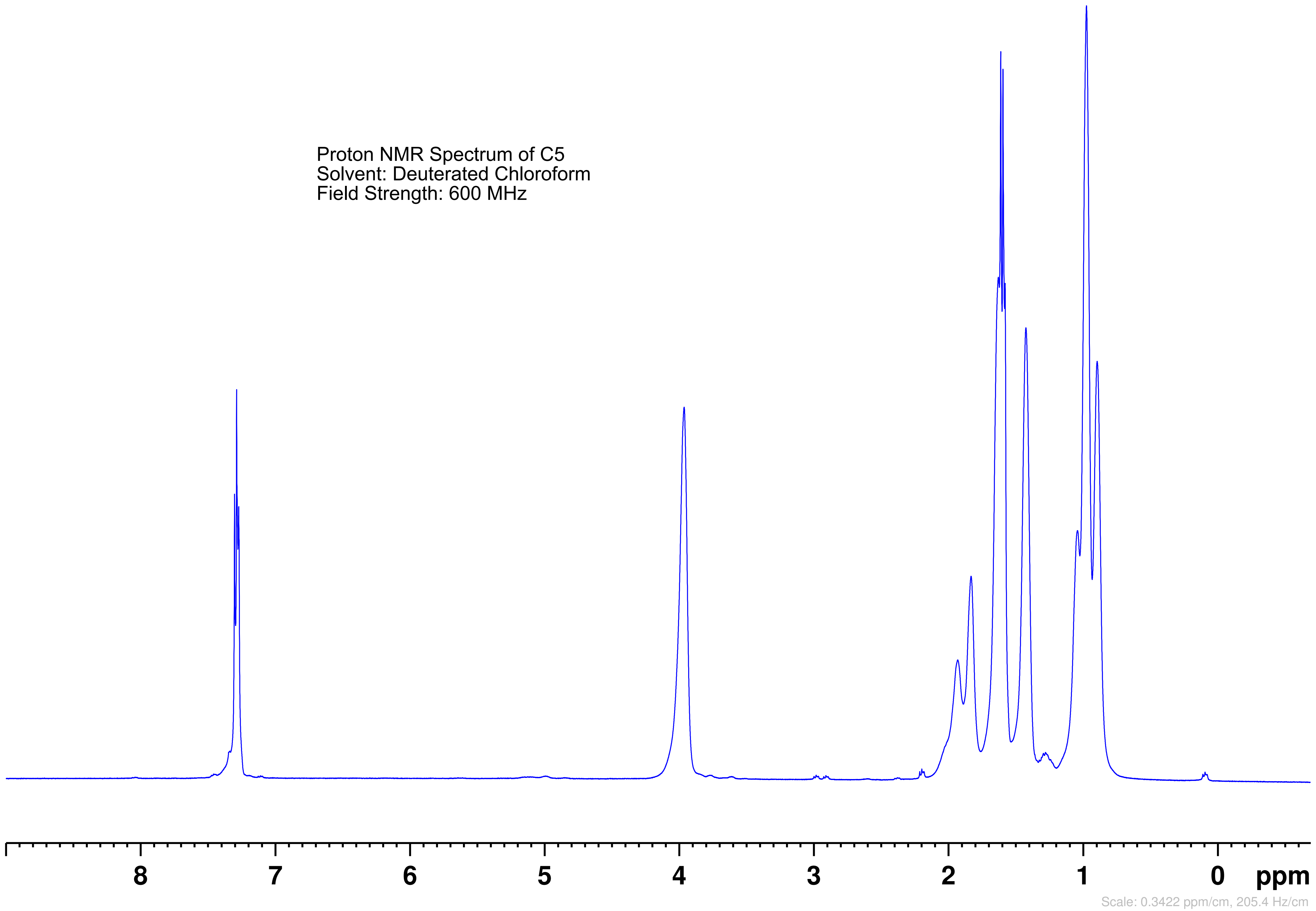

Proton NMR Spectrum of C5
Solvent: Deuterated Chloroform
Field Strength: 600 MHz
8
7
6
5
4
3
2
1
0
ppm
Scale: 0.3422 ppm/cm, 205.4 Hz/cm
